# Regulatory Dendritic Cells Induced by K313 Display Anti-Inflammatory Properties and Ameliorate Experimental Autoimmune Encephalitis in Mice

**DOI:** 10.3389/fphar.2019.01579

**Published:** 2020-01-28

**Authors:** Yan Zhou, Xiao Leng, Xingyan Luo, Chunfen Mo, Qiang Zou, Yang Liu, Yantang Wang

**Affiliations:** ^1^ Department of Emergency, West China Second University Hospital and Key Laboratory of Obstetric and Gynecologic and Pediatric Diseases and Birth Defects, Ministry of Education, Sichuan University, Chengdu, China; ^2^ Department of Pharmacology, School of Pharmacy, Chengdu Medical College, Chengdu, China

**Keywords:** benzoxazole derivative, regulatory/tolerogenic dendritic cells, GSK-3β, K313, experimental autoimmune encephalitis/multiple sclerosis

## Abstract

As a GSK-3β inhibitor reported by our group, K313 is a novel benzoxazole derivative and displays anti-inflammatory properties in RAW264.7 macrophages without cytotoxicity. The activity of GSK-3β affects the differentiation and maturation of bone marrow-derived dendritic cells (DCs). This study aims to investigate whether K313 can be used to induce regulatory/tolerogenic dendritic cells (DCregs), and the therapeutic effects of DCregs induced by K313 in the autoimmune model of experimental autoimmune encephalitis (EAE). The results show that compared with LPS stimulated mature DCs, K313-treated bone marrow-derived DCs display obvious tolerogenic characteristics with decreased expression of co-stimulatory molecules, downregulated secretions of pro-inflammatory cytokines and unregulated secretion of anti-inflammatory cytokine IL-10. The above characteristics conform to the typical phenotypes of DCregs. Moreover, K313-modified DCregs inhibit antigen-specific T cell responses *in vitro*. Furthermore, by adoptive transfer, K313 modified DCregs to the EAE mice, and the development of disease was ameliorated to some extent. In addition, treatment with K313-modified DCregs also significantly reduced the percentages of splenetic Th1 and Th17 cells and increased the percentage of regulatory T cells in EAE mice. In conclusion, K313-modified DCregs show anti-inflammatory properties *in vitro* and have a significant positive effect on the EAE disease *in vivo*. Our data indicate that K313-induced DCregs pulsed with auto-antigen might have potential use as a therapeutic approach for autoimmune inflammation of the central nervous system.

## Introduction

Dendritic cells (DCs) play an important role in bridging innate and adaptive immunity. On the one hand, through recognizing danger signals from outer stimuli or injured host cells, DCs differentiate into mature DCs, which present antigens to prime naïve T cells for generating adaptive immune responses. On the other hand, regulatory/tolerogenic dendritic cells (DCregs) exert key roles in the regulation of the immune system through the induction of T cell anergy, clearance of auto-reactive T cells, and immune tolerance (Qian and Cao, 2018). Compared with mature DCs, DCregs, which are characterized by reduced co-stimulatory molecules and pro-inflammatory cytokines, can attenuate pro-inflammatory T cell-priming and promote regulatory T cells (Tregs) (Stojanovic et al., 2017). Based on the immune tolerance function, the adoptive transfer of DCregs pulsed with auto-antigens is a potential therapeutic method for autoimmune disorders (Phillips et al., 2017). Multiple sclerosis (MS) and the animal model of this disease named experimental autoimmune encephalitis (EAE) is an autoimmune disease that displays inflammatory demyelinating, axonal injuring, and neurological impairment in the central nervous system (Benkhoucha et al., 2010). As the first line relapsing-remitting MS treatment drug, type I IFN and glatiramer acetate have been widely used for a long time (Westad et al., 2017). However, current treatment has unpleasant side effects, including itching, alopecia, and liver toxicity (Barbero et al., 2006). Encouragingly, there is evidence to suggest that DCregs pulsed with auto-antigens are effective and safe to treat a variety of autoimmune disorders (Phillips et al., 2019). Currently, some immunosuppressive agents and natural products are suggested to effectively generate DCregs, and the compounds that modify DCregs can assist in the improvement of disease in EAE mice. The DCregs generated with 1,25-dihydroxyvitamin D3 or vitamin D3 can attenuate EAE by suppressing infiltrations of auto-reactive T cells, such as Th1 and Th17 subsets, in the spinal cord (Piemonti et al., 2000; Sinha et al., 2010; Domogalla et al., 2017). Natural products, such as Apigenin and Estriol, have also been found to induce DCregs, which play a therapeutic function in EAE mice (Papenfuss et al., 2011; Ginwala et al., 2016). Our previous findings suggested that tolerogenic DCs modified by the benzothiazole derivative BD750 can ameliorate EAE in mice, and a similar result was found by our group for tofacitinib-induced tolerogenic DCs (Zhou et al., 2016; Zhou et al., 2017). Though DCregs therapy is a potential method for the treatment of EAE and MS, several problems still require solving. Not only do the injection dose, route, and frequency need to be optimized, but searching for new agents to induce DCregs with stable tolerogenic phenotypes is another challenge (Stojanovic et al., 2017). As reported above, searching for new immunosuppressive agents to induce DCregs for the treatment of autoimmune diseases with higher safety and clinical efficacy is crucial.

Bone marrow-derived dendritic cells (BMDCs), which differentiate from murine bone marrow cells and are cultured with granulocyte-macrophage colony-stimulating factor (GM-CSF), are widely used for DCregs research (Phillips et al., 2017). As per recent reports, cultured BMDCs stimulated with GM-CSF are a mixture of cell populations that consist of monocyte-derived macrophages and conventional DCs. The two groups can be distinguished according to the expression of surface markers, such as CD11c and MHC II (Helft et al., 2015). Yi Rang Na et al. suggested that conventional DCs show higher MHC II and CD11c+, but lower F4/80 phenotypes compared with monocyte-derived macrophages. Meanwhile, the proportion of macrophages in cultures was positively correlated with the concentration of GM-CSF (Na et al., 2016). The above studies have demonstrated that DCs have a greater ability to stimulate T cell proliferation than monocyte-derived macrophages (Helft et al., 2015; Na et al., 2016); therefore, acquiring pure BMDCs is vital to DCregs preparation.

K313 is a novel benzoxazole derivative [1H-indole-2,3-dione 3-(1,3-benzoxazol-2-ylhydrazone)], our previous study shows that this small molecule has an anti-inflammatory effect on RAW264.7 macrophages which are treated with LPS by enhancing phosphorylated GSK-3β (Ser9) levels to inactivate GSK-3β (Zhao et al., 2018). Glycogen synthase kinase 3 (GSK-3) is a serine/threonine kinase which consists of two isoforms: GSK-3α and GSK-3β. Recent studies have demonstrated that GSK-3β plays a crucial role in the differentiation and maintenance of immature mouse DCs (imDCs) and human monocyte-derived DCs (Menon et al., 2007). Martin *et al.* have suggested that the inhibition of GSK-3β could upregulate IL-10 levels and decrease the secretions of IL-12p40, IL-6, and TNF-α in human monocytes (Martin et al., 2005). In addition, BMDCs treated with a specific GSK-3β inhibitor displayed immature phenotypes with reduced surface markers, such as CD40, CD80, CD86, and MHC II, and the agent-treated DCs secreted lower IL-12 and higher IL-10 (Ono et al., 2007). Furthermore, as a GSK-3 inhibitor, lithium chloride (LiCl) has been used to treat EAE in animal models and displays a strong inhibitory capacity for inflammation (Kim et al., 2015). Therefore, this study aimed to determine the tolerogenic ability of K313 modified DCs, and the new DCregs generation method may provide a potential therapeutic avenue for the treatment of autoimmune diseases, including MS.

## Materials and Methods

### Animals

Female C57BL/6 mice (6–8 weeks) were purchased from the Vital River Laboratory Animal Technology Corporation (Beijing, China). The OT-II TCR transgenic mice were a gift from Guixiu Shi (University of Xiamen, China). All mice were bred in the specific pathogen-free facility of Chengdu Medical College, and the experimental protocols were approved by the Animal Care and Use Committee of Chengdu Medical College. All experimental animal protocols were followed regarding the national requirements for animal ethics.

### Murine Bone Marrow-Derived Dendritic Cells Cultured and Treated With K313

**Figure 1:**
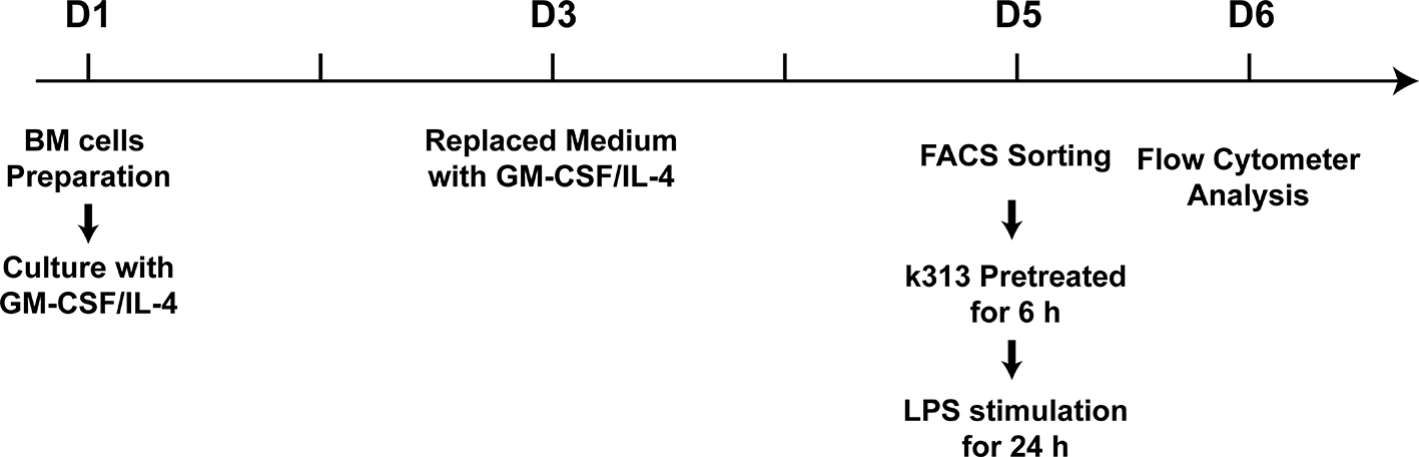
The cartoon of time points for *in vitro* experiments.

Female C57BL/6 mice (6–8 weeks) were anesthetized and euthanized by cervical disconnection. The femur and tibia bones were isolated aseptically, and then washed once with 75% alcohol, and three times with cold phosphate-buffered saline (PBS). After the ends of the bones were cut, a 1 ml sterile syringe was used for flushing out the bone marrow cells with 5 ml of cold PBS. Then, the cell suspensions were passed through a nylon mesh to remove small pieces of bone and debris. Subsequently, the single bone marrow cells were washed with cold PBS, and 1 × 10^7^ cells were plated in 10 ml RPMI 1640 medium containing 10% FBS, penicillin, and streptomycin supplemented with 20 ng/ml recombinant murine GM-CSF and 10 ng/ml recombinant murine IL-4 (PeproTech). Then, half of the medium was displaced every 2 days. On day 5, the cells were collected, and CD11c+MHCII+ DCs were sorted using a BD FACSJazz cell sorter (BD Biosciences). The sorted cells were plated in a 24-well plate and treated with 1, 4, and 16 μM K313 (# 5939009, ChemBridge Corp, San Diego, CA, USA) (**Figure 1**), and DMSO-treated cells were used as vehicle control. After 6 h, 100 ng/ml LPS was added to stimulate the maturation of BMDCs.

### Human Dendritic Cells Cultured and Treated With K313

Ethical approval was obtained through the Ethical Review Committee of Chengdu Medical College, and informed consent of all participating subjects was obtained. The protocol of generating human DCs from human blood mononuclear cells [peripheral blood mononuclear cells (PBMCs)] has been described (Nair et al., 2012). In short, peripheral blood was directly drawn into vacuum blood collection tubes containing sodium heparin, and PBMCs were isolated using a density gradient centrifugation on Ficoll-Paque Plus solution (Dakewei, Beijing, China). CD14+ monocytes were sorted using a BD FACSJazz cell sorter and cultured in RPMI 1640 medium containing 10% FBS, penicillin, and streptomycin, supplemented with 40 ng/ml recombinant human GM-CSF and 20 ng/ml recombinant human IL-4 (PeproTech) for 5 days. The culture medium, including supplements, was refreshed on day 3. On day 5, the cells were plated in a 24-well plate and treated with 1, 4, and 16 μM K313. DMSO-treated cells were used as vehicle control. After 6 h, 200 ng/ml LPS was added to stimulate the maturation of DCs.

### Flow Cytometry Analysis

On day 6, the cultured cells were collected and washed once with cold PBS. After blocking with anti-mouse CD16/CD32 (Mouse BD Fc Block, BD Pharmingen) or human BD Fc Block (BD Pharmingen), the cells were labeled with the following antibodies: PE-Cy7-anti-mouse CD11c (clone: HL3), FITC-anti-mouse CD40 (clone: 3/23), FITC-anti-mouse CD80 (clone: 16-10A1), PE-anti-mouse CD86 (clone: GL-1), Alexa Fluor647-anti-mouse CCR7 (clone: 4B12) (BioLegend) or APC-anti-human CD80 (clone: 2D10), PE-anti-human CD86 (clone: BU63), FITC-anti-human CD40 (clone: 5C3), BV421-anti-human CD14 (clone: MΦP9). After staining on ice for 30 min and washing once with cold PBS, the cells were detected by a flow cytometer (ACEA Biosciences Quanteon NovoCyte) and the data were analyzed by ACEA Novo Express software.

To detect the survival of K313-treated BMDCs, the apoptosis of cells from the culture on day 6 was analyzed by flow cytometry using a PE-Annexin V Apoptosis Detection Kit I (BD Pharmingen) according to the corresponding protocol; cells were cultured and treated with K313 as described above (Method 2.2). To test whether the phagocytosis of DCs was influenced by K313, the CD11c+MHC II+ DCs pretreated with K313 were prepared as per the previous method. FITC-Dextran (40,000 Da, Sigma) was incubated with the cells at 37°C for 30 min and stopped by cold PBS. Cells incubated in FITC-Dextran solution at 4°C were used as a negative control. Then, the cells were washed once and analyzed using a flow cytometer.

### *In Vitro* Lymphocyte-Proliferation Assays

The sorted BMDCs were cultured and treated as per the above method. Finally, the prepared cells were pulsed with OVA_323-339_ for 1 h. Meanwhile, splenic cells were isolated from female OT-II mice (6–8 weeks). After a grinding step, red blood cell lysis buffer was added to remove the red blood cells; then, the cells were washed twice with cold PBS to prepare the single-cell suspension. The naïve CD4+ T cells were purified using a Naive CD4+ T Cell Isolation Kit (Miltenyi), according to the manufacturer's instructions. Subsequently, the purified cells were stained with carboxyfluorescein diacetate succinimidyl ester (CFSE) (Invitrogen) following the standard protocols, and then co-cultured with OVA_323-339_ pulsed BMDCs in a 24-well plate for stimulating antigen-specific T cell proliferation. The ratios of DCs: T was 1:10, 1:30, and 1:100, the number of T cells was the same in each groups, with a proportional change in the number of DCs. After 5 days, the cells were collected, stained with CD4-PE, and detected using a flow cytometer.

### Detection of Cytokine Production in Cultural Supernatants

The sorted BMDCs were treated and cultured as per the above method. On day 6, the culture medium was collected and centrifuged at 300 g at 4°C for 5 min. The supernatants were gathered for the ELISA test. IL-12, IL-6, IL-10, and TNF-α were measured according to the protocols of the ELISA Kits (BD Pharmingen).

### Experimental Autoimmune Encephalitis Induction and Adoptive Transfer of K313-Modified Regulatory/Tolerogenic Dendritic Cells

Female C57BL/6 mice (6–8 weeks) were immunized subcutaneously with 200 μg of MOG_35–55_ peptide, as previously described (Zhou et al., 2017). Each mouse was injected with 500 ng pertussis toxin (PTx) *i.p.* on days 0 and 2. The culturing method of BMDCs was conducted as per the above method. On day 5, the sorted DCs were pretreated with K313 (16 μM) for 6 h, then MOG_35-55_ (20 μg/ml, Bankpeptide, Hefei, China) and LPS (100 ng/ml) were added to the treated cells for 24 h. The cells were washed and transferred into the EAE mice *via* tail vein injection (1 × 10^6^ cells/mouse) on days 10, 13, and 16. DCregs induced by 1, 25-Dihydroxyvitamin D_3_ (1,25-D3) (10^–8^ M; Sigma-Aldrich) were used as a positive control. The development of EAE was observed daily, and clinical scores were made according to the Benson score: 0, no clinical signs; 1, paralyzed tail; 2, loss of coordinated movement, hind limb paresis; 3, both hind limbs paralyzed; 4, forelimbs paralyzed; and 5, moribund (Zhou et al., 2017).

### Histological Analysis

On day 20, the three groups of mice were anesthetized with sodium pentobarbital and perfused with PBS (pH 7.4) and 4% (w/v) paraformaldehyde. Subsequently, 4% (w/v) paraformaldehyde was used to fix spinal cord samples overnight. Through paraffin embedding, slicing, and dewaxing hydration, the coronal sections (5 μm) were stained with hematoxylin and eosin (HE) to examine the extent of cellular infiltration. Meanwhile, the luxol fast blue staining (LFB) method was used to determine demyelination.

### Intracellular Staining of CD4+ T Cells

The functional phenotypes of splenetic CD4+ T cells from EAE mice were analyzed by flow cytometry. Briefly, splenocytes were treated with anti-mouse CD16/CD32 (Mouse BD Fc Block, BD Pharmingen) and stained with FITC-anti-CD4 (clone:RM4-5), fixed, permeabilized (BD Cytofix/Cytoperm Fixation/Permeabilization Solution Kit, BD Pharmingen), and intracellularly stained with PE-anti-IFN-γ (clone: XMG1.2) and Alexa Fluor647-anti-IL-17 (clone: TC11-18H10), followed by flow cytometer (ACEA Biosciences Quanteon NovoCyte). Some splenocytes were stained with FITC-anti-CD4 and APC-anti-CD25 (clone: PC61) (BD Pharmingen), fixed, permeabilized (Transcription Factor Buffer Set, BD Pharmingen), and stained with PE-anti-Foxp3 (clone: R16-715) (BD Pharmingen), followed by flow cytometer analysis of the frequency of Tregs.

### Statistical Analysis

The data are expressed as the mean ± SD. Differences between groups were analyzed by repeated analysis of variance (ANOVA) and *post hoc* Bonferroni correction. All statistical analyses were performed using PRISM 6.0 software (GraphPad Software). A *P* value of < 0.05 was considered statistically significant.

## Results

### K313 Did Not Affect Phagocytosis or Survival of Dendritic Cells

**Figure 2:**
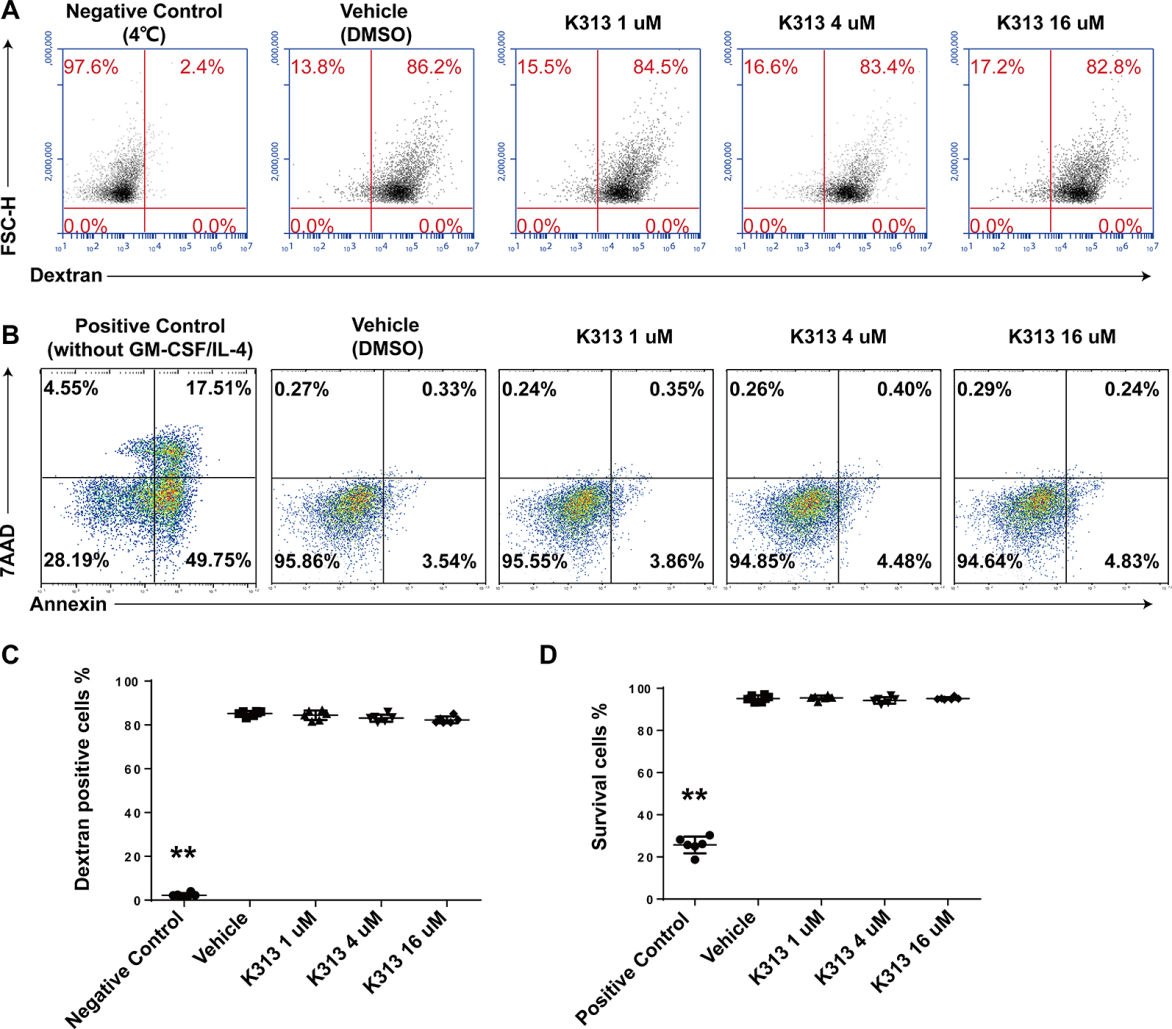
K313 had no effect on phagocytosis or survival of DCs. **(A)** The sorted murine DCs were collected and treated with 1, 4, and 16 μM K313 for 6 h, then incubated with FITC-dextran at 4 or 37°C for 30 min and immediately stopped by cold PBS, finally washed once and analyzed by FACS. **(B)** The sorted DCs was collected and treated with 1, 4, and 16 μM K313. After culturing for 24 h, cells were collected and stained with PE-Annexin V and 7-AAD followed the protocol of the kit, finally analyzed by flow cytometer. **(C)** The phagocytosis of DCs was assessed by the proportion of FITC-dextran positive cells, each column denoted the mean ± SD of six independent experiments results (*P < 0.05, **P < 0.01 versus the vehicle group). **(D)** Cells with double negative of staining with PE-Annexin V and 7-AAD represented the survival cells (Annexin V-7-AAD- cells), each column denoted the mean ± SD of six independent experiments results (*P < 0.05, **P < 0.01 versus the vehicle group).

Phagocytosis is an important characteristic of imDCs. In the pathological process of autoimmunity, DCs recognize and uptake the self-antigens to initiate autoimmune inflammation (Wang et al., 2018). Therefore, whether K313 influenced the phagocytosis of imDCs was tested. The cells were exposed to 1, 4, and 16 μM of K313, while the vehicle group was blank. Results showed that there is no difference in the proportion of FITC-dextran-positive cells between the K313 and vehicle groups. K313 does not affect the phagocytosis of imDCs, even at the highest concentrations of 16 μM (**Figures 2A, C**). Meanwhile, the Annexin V-PE/7-AAD double dyeing was performed to evaluate the apoptosis of DCs stimulated with LPS for 24 h. As shown in **Figures 2B, D**, K313 did not affect the survival rate of DCs at any concentration, compared to the vehicle group. However, the GM-CSF/IL-4 starved group showed a marked increase in the proportions of early apoptotic cells (Annexin V+ 7-AAD-), and K313 treated DCs showed obvious apoptosis following LPS stimulation for 48 h (**Supplementary Figure 1**). In the following *in vitro* and *in vivo* experiments, the period of LPS stimulation for DC maturation was 24 h. The low cytotoxicity of K313 is evidenced by the above data which is consistent with our previous study (Zhao et al., 2018).

### K313 Inhibited Maturation of Dendritic Cells Stimulated With LPS

**Figure 3:**
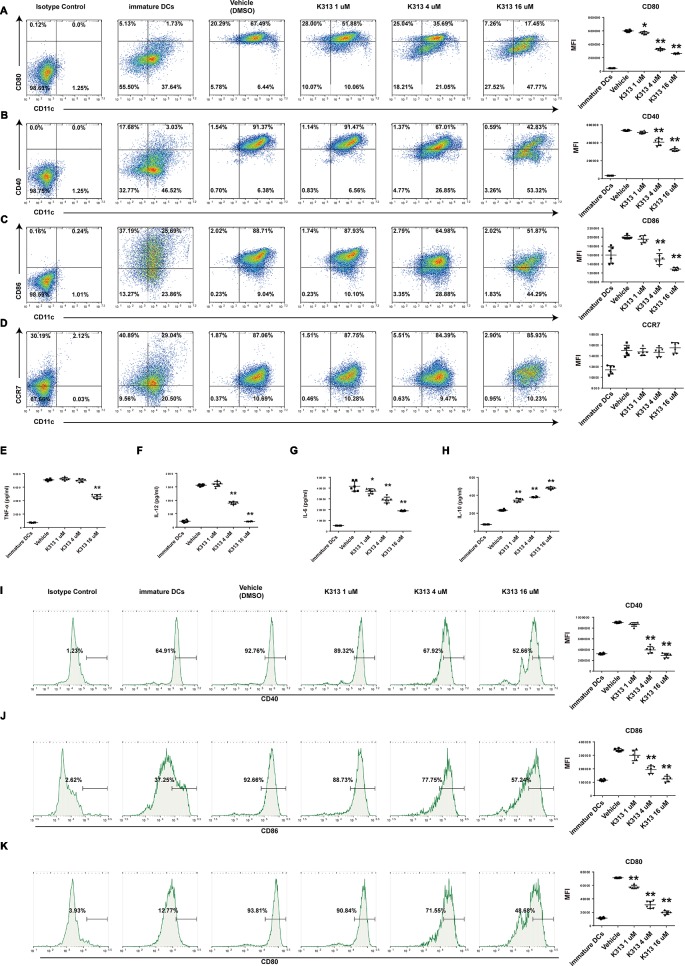
K313 had the ability to suppress maturation of DCs. The sorted murine DCs were collected and treated with 1, 4, and 16 μM K313, LPS was added after 6 h. After culturing for 24 h, cells were collected and washed, then stained with indicated antibodies such as anti-murine CD11c, anti-murine CD80, anti-murine CD40, anti-murine CD86, and anti-murine CCR7, finally analyzed by flow cytometer. Flow cytometry analysis of anti-murine CD80 **(A)**, anti-murine CD40 **(B)**, anti-murine CD86 **(C)**, and anti-murine CCR7 **(D)** expressions on murine DCs treated with K313. Histogram analysis of mean fluorescence intensity (MFI). The supernatants were gathered for ELISA test. The cytokines such as TNF-α **(E)**, IL-12 **(F)**, IL-6 **(G)**, and IL-10 **(H)** were measured according to protocols of ELISA Kits which purchased from BD Pharmingen. Histogram analysis of each cytokine expression. Human PBMCs derived DCs were collected and treated with 1, 4, and 16 μM K313, LPS was added after 6 h. After culturing for 24 h, cells were collected and washed, then stained with indicated antibodies such as anti-human CD14, anti-human CD40, anti-human CD86, and anti-human CD80, finally analyzed by flow cytometer. Flow cytometry analysis of anti-human CD40 **(I)**, anti-human CD86 **(J)** and anti-human CD80 **(K)** expressions on human DCs treated with K313. Histogram analysis of MFI, each column denoted the mean ± SD of six independent experiments results (*P < 0.05, **P < 0.01 versus the vehicle group).

Regulatory DCs exhibited immature phenotypes of lower expression of specific surface markers compared with mature DCs (Hawiger et al., 2001). Of the tested surface markers, CD40, CD80, and CD86, which are co-stimulatory molecules, are indispensable for initiating antigen-specific T cell activation (Rescigno et al., 1998). Therefore, we investigated CD40, CD80, CD86, and CCR7 expression through flow cytometry to evaluate whether K313 affects the maturation of murine and human DCs stimulated with LPS. The results show that the CD40, CD80, and CD86 of murine DCs treated with K313 (4 and 16 μM) decreased, compared with the vehicle group (**Figures 3A–D**). Furthermore, the amount of above-mentioned surface markers gradually decreased, with an increase in K313 concentration (**Figures 3A–C**). Moreover, the expression of CD80, CD86, and CD40 in the 16 μM K313 group was drastically reduced by 66, 41, and 53%, respectively, compared with the vehicle group (**Figures 3A–C**). Similarly, maturation markers (CD40, CD86, and CD80) of human DCs treated with k313 at concentrations of 4 and 16 μM also displayed significantly reduced levels relative to the vehicle control (**Figures 3I–K**). Collectively, the DCs treated with K313 displayed obvious down-regulation of co-stimulatory molecules.

### K313-Treated Bone Marrow-Derived Dendritic Cells Secreted Increased IL-10 and Decreased Pro-Inflammatory Cytokines

To investigate whether K313 affects the cytokine secretions of BMDCs, the amounts of pro-inflammatory cytokines, such as IL-12, IL-6, TNF-α, and anti-inflammatory cytokine IL-10, were measured using ELISA Kits. The results indicate that the K313-treated DCs secreted increased IL-10 compared with the vehicle group, and the increased expression of IL-10 was positively correlated to the K313 concentration (**Figure 3H**). In contrast, the amount of secreted IL-12 dramatically decreased, and the secretion levels of the cytokine dropped by about seven-fold when DCs were treated with 16 μM K313 (**Figure 3F**). An increased ratio of secreted IL-10: IL-12 was a typical tolerance characteristic of DCregs (Kouwenberg et al., 2016). In keeping with previous findings, DCs treated with 16 μM K313 exhibited a significantly higher ratio of IL-10: IL-12 compared to the vehicle group. In addition, this study showed that the secretion of TNF-α was down-regulated by 16 μM K313 (**Figure 3E**), and treatment with k313 at a higher dose significantly reduced the levels of LPS-stimulated IL-6 (**Figure 3G**). These results suggest that 16 μM K313 can effectively elevate the levels of IL-10, in contrast, to the suppression of the production of IL-12, IL-6, and TNF-α in culture supernatant of BMDCs.

### K313-Treated Bone Marrow-Derived Dendritic Cells Modulate Antigen-Specific T Cell Responses

**Figure 4:**
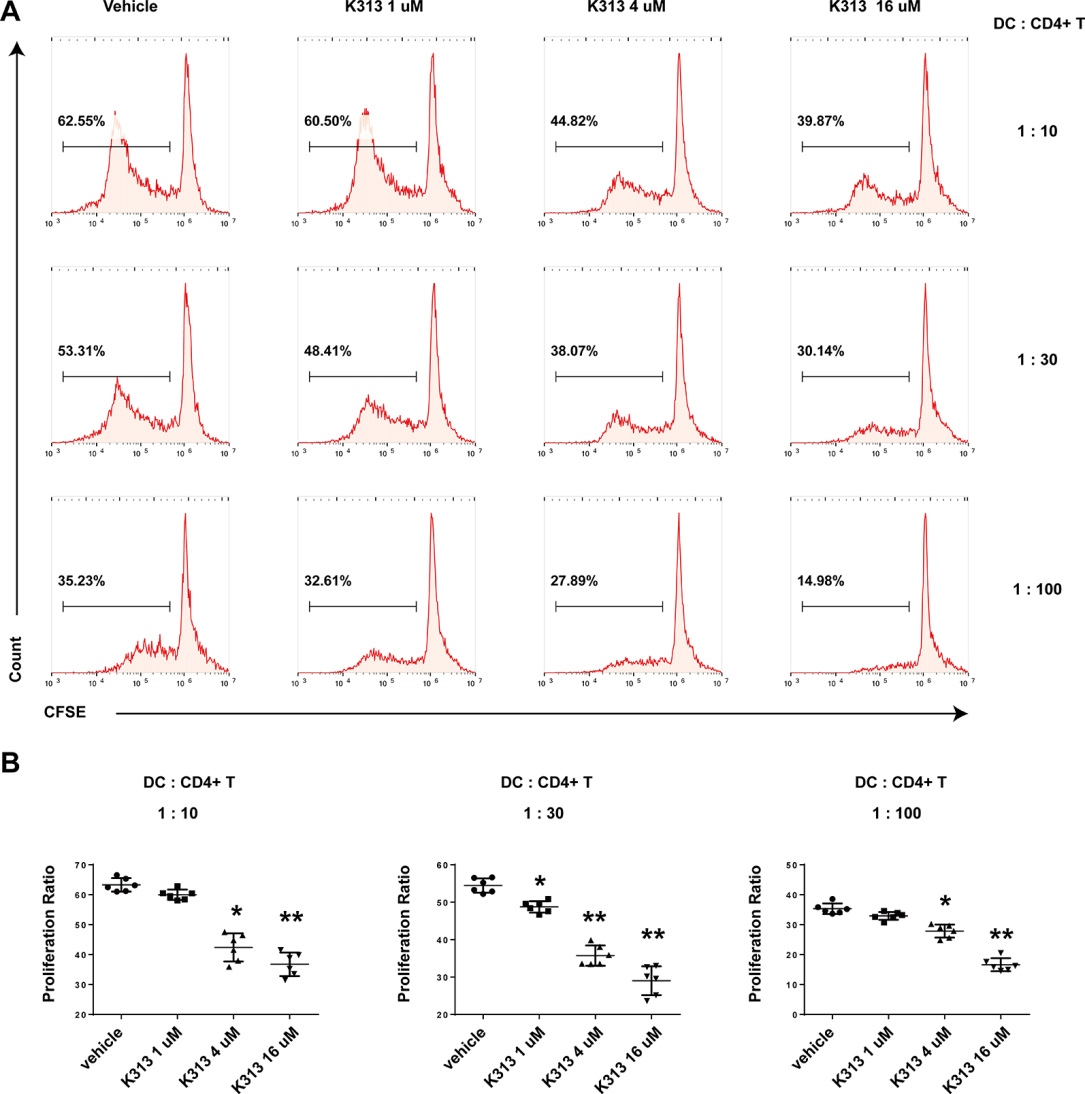
K313 modified DCregs suppressed the proliferation of OVA_323-339_ peptide-specific CD4+ T cells. The splenic cells were obtained from OT-II mice, naïve CD4+ T cells were separated and stained with CFSE, then co-cultured with K313 modified DCregs pulsed with OVA_323-339_ in three ratios of DCs:T (1:10, 1:30, 1:100) separately for 5 days, finally stained with PE-CD4 and analyzed by flow cytometer. **(A)** Proliferation activities of OVA_323-339_ peptide-specific CD4+ T cells were determined by flow cytometry analysis. **(B)** Histogram analysis of proliferation rate at each DCs:T ratio separately, each column denoted the mean ± SD of six independent experiments results (*P < 0.05, **P < 0.01 versus the vehicle group).

The ability to inhibit autoreactive T cells is an important characteristic of DCregs (Nishimura et al., 2015). In order to test the influence of K313 on the proliferation of antigen-specific CD4+ T cells primed by DCs, K313-modified DCregs were pulsed with OVA_323-339_ for 1 h and then co-cultured with purified splenic naïve CD4+ T cells from OT II mice at various DCs: T ratios for 5 days. Finally, the proliferation rate of CFSE-labeled CD4+ T cells was tested by flow cytometry. As expected, the proliferation rate of antigen-specific T cells was positively correlated with the DCs: T ratio and the T cells in the vehicle group showed the most expanded ability in each ratio (**Figure 4A**). Meanwhile, with the increase of K313 treatment concentration in DCs, the proliferation rate of antigen-specific CD4+T cells exhibited a gradual downward trend. (**Figure 4B**). The results suggest that BMDCs treated with K313 can suppress the proliferation of OVA_323-339_ peptide-specific CD4+ T cells.

### K313-Modified Regulatory/Tolerogenic Dendritic Cells Loaded With MOG_35-55_ Ameliorated Experimental Autoimmune Encephalitis in Mice

**Figure 5:**
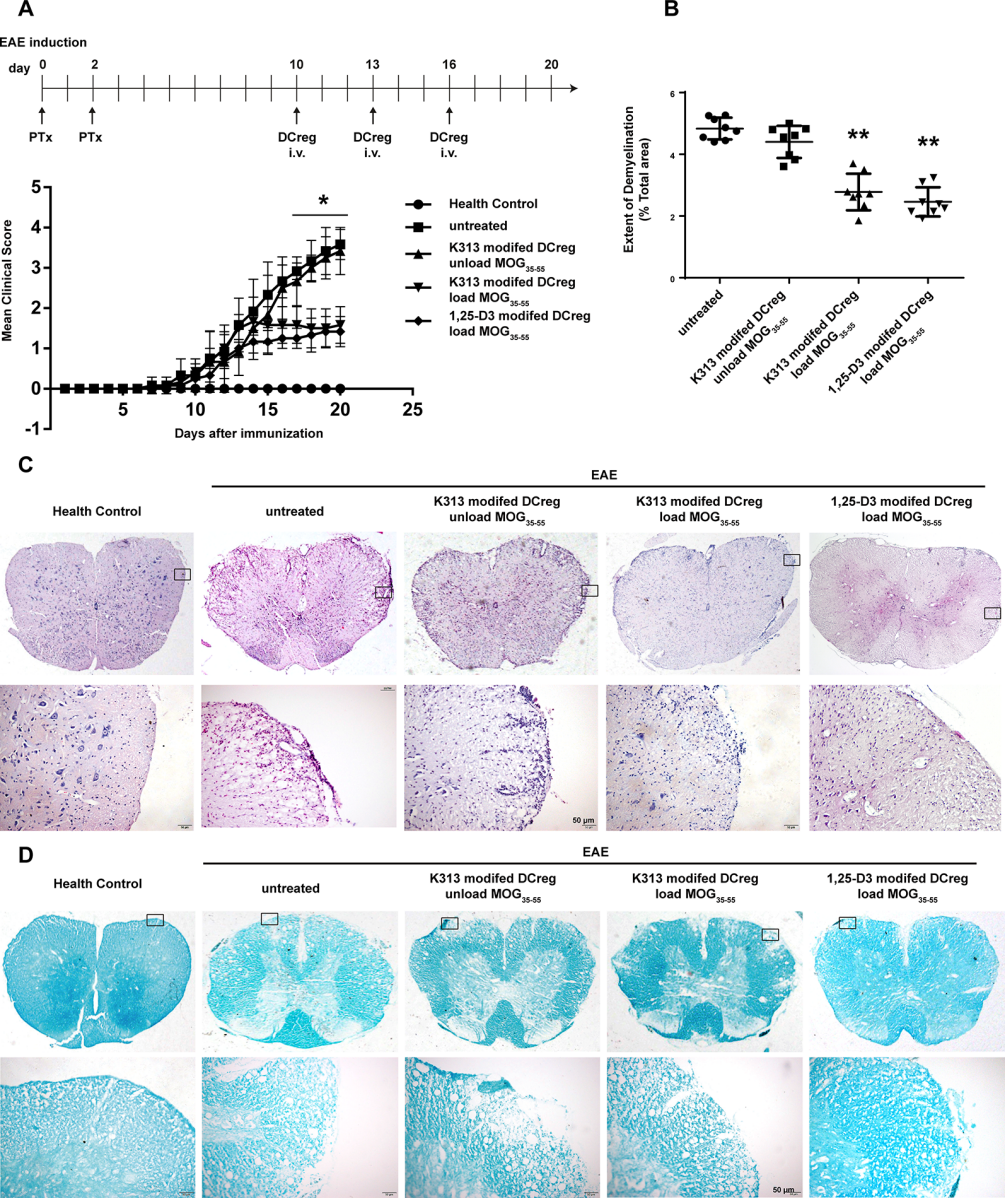
Adoptive transfer K313 modified DCregs loaded with MOG_35-55_ into the EAE mice ameliorated the development of disease. The 16 μM K313 modified DCregs loaded or unloaded with MOG_35-55_ (20 μg/ml) were transferred into the EAE mice by intravenous injection (1 × 10^6^ cells/mouse) at day 10, 13, and 16 postimmunization. The positive control group were given 10^–8^ M 1,25-Dihydroxyvitamin D_3_ induced DCregs loaded with antigen. The clinical scores were made according to Benson score. On day 20, the five groups of mice were perfused. After spinal cord sample fixation, paraffin embedding, slicing and dewaxing hydration, HE and LFB staining were performed. **(A)** The mean clinical scores of five groups of mice. The experiment was performed twice, the total number of animals in each group was 8 (n = 8). **(B)** Quantitative comparison of the extent of demyelinated area in different animal groups. 24 sections from each group were analyzed by ImageJ software for assessing the myelination intensity and the extent of demyelinated area. (*P < 0.05, **P < 0.01 versus the untreated group). **(C)** Histological examinations staining examined the extent of cellular infiltration. **(D)** LFB staining examined the extent of demyelinating (magniﬁcation: 200×).

**Figure 6:**
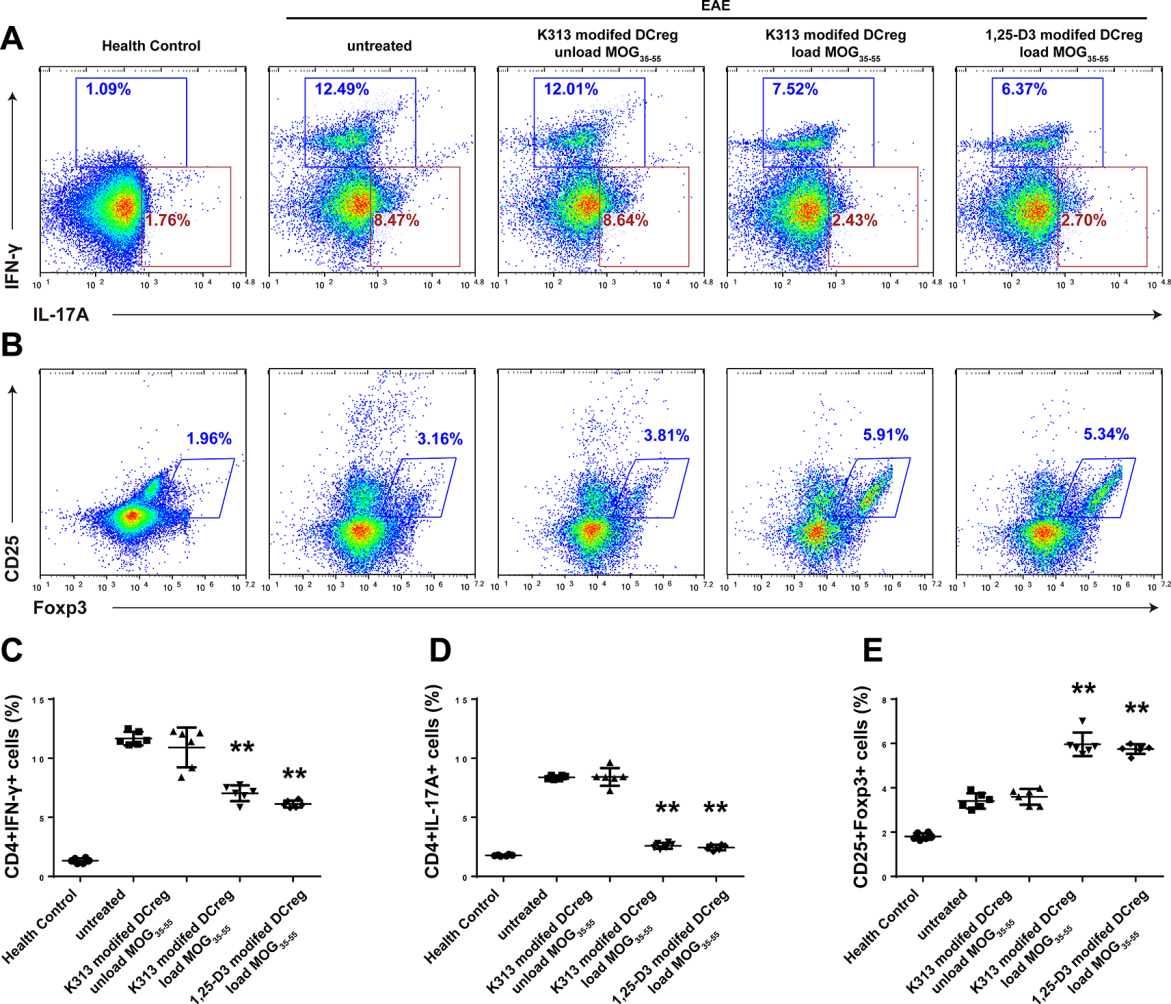
Adoptive transfer of K313-treated DCregs disturbed the balance among Th1, Th17 cells and Tregs in EAE mice. On day 20 post the first PTx injection, some mice from each group were euthanized and their splenic T cells were stained with anti-CD4/anti-CD25, intracellularly stained with anti-IL-17A, anti-IFN-γ or anti-Foxp3. **(A**–**B)** The percentages of splenic Th1 and Th17 cells and Tregs were determined by flow cytometry. **(C**–**E)** Histogram analysis of intracellular marker expression, each column denoted the mean ± SD of each group of mice (n = 6 per group) (*P < 0.05, **P < 0.01 versus the untreated group).

The results of our experiments *in vitro* show that K313 can induce DCregs, and K313-modified DCregs can suppress the proliferation of antigen-specific CD4+ T cells. To investigate whether DCregs modified by the agent have a therapeutic effect on animal models of autoimmune diseases, K313-modified DCregs loaded or unloaded with MOG_35-55_ were transferred into EAE mice through tail vein injection. The results indicate that the group of antigen-loaded DCregs had a milder clinical score than the antigen-unloaded group, and the therapeutic effects of K313-modified DCregs were similar to that of 1, 25-Dihydroxyvitamin D_3_-induced DCregs (**Figure 5A**). In addition, HE staining showed that the degree of leukocyte infiltration was dramatically reduced in the antigen-loaded DCregs group (**Figure 5C**), and LFB staining also indicated that there was extensively decreased demyelination in the MOG_35-55_-loaded DCregs group (**Figures 5B, D**). Overall, by the adoptive transfer of K313-modified DCregs loaded with MOG_35-55_, the development of disease in EAE mice was ameliorated to some extent. To understand the underlying mechanisms by which the direct cell-cell interaction between adoptive transferred DCs and T cells exacerbated the development of EAE, the percentages of splenetic Th1 and Th17 cells and Tregs in the different groups of mice were characterized by flow cytometry. The results show that adoptive transfer with K313-modified DCregs loaded with antigens significantly reduces the percentages of Th1 and Th17 cells and increases the percentage of Tregs compared with the untreated group (**Figure 6**).

## Discussion

Antigen peptide-loaded DCregs are effective in treating models of autoimmune disorders due to their tolerogenic characteristics and ability to suppress the responses of antigen-specific T cells (Zhou et al., 2016; Zhou et al., 2017). For clinical treatment, the agents used for DCreg induction should be safe, efficient, and have low toxicity. It has been suggested that K313 has no influence on the phagocytosis and survival of DCs in this study, and our previous reports also indicated that K313 has no cytotoxic effect on RAW264.7 macrophages in CCK-8 assays (Zhao et al., 2018). More importantly, K313 has been shown to exhibit an anti-inflammatory function in LPS-stimulated RAW264.7 macrophages by inhibiting the activity of GSK-3β (Zhao et al., 2018). GSK-3β was found to play an important role in LPS-induced DC maturation through the stimulation of TLR4 (Ono et al., 2007). Subsequently, the PI3K/AKT pathway was activated, and the activated AKT inhibited the activity of GSK-3β by serine phosphorylation. The inactivated GSK-3β finally interfered with the production of co-stimulatory molecules and the cytokine secretions in DCs (Pap and Cooper, 1998). Therefore, this study aimed to investigate whether K313 could shape the tolerogenic phenotype of DCs and influence the proliferation of antigen-specific CD4+ T cells primed by this agent's modified DCs. The experimental results showed that K313-treated BMDCs decreased the production of co-stimulatory molecules, upregulated the secretion of anti-inflammatory cytokine IL-10, downregulated secretions of pro-inflammatory cytokines, and reduced the priming of antigen-specific CD4+ T cells. Furthermore, the adoptive transfer of antigen peptide-loaded DCregs into EAE mice ameliorated the development of disease to some extent, through reducing the percentages of pathogenic Th1/Th17 cells and increasing the percentage of Tregs.

Through interactions with CD28 on T cells, CD80 and CD86 are considered as important co-stimulatory molecules of antigen-presenting cells for T cell priming and IL-2 production (Schwartz, 1992). CD40 was another co-stimulatory molecule of DCs; the cytokine functioned as the modulating secretion of Th1/Th2 cytokine expression levels (Peng et al., 1996). The decreased amounts of CD80, CD86, and CD40 were typical phenotypes of DCregs, compared with mature DCs (Rulifson et al., 1997). Ono *et al.* found that inhibition of GSK-3β activity could downregulate the expression of CD40, CD80, CD86, and MHCII on BMDCs (Ono et al., 2007). Kouwenberg *et al.* also suggested that the inhibition of GSK-3, together with triggering TLR2 could generate tolerogenic DCs for decreased CD40 and CD86 (Kouwenberg et al., 2016). Consistent with previous studies, the new GSK-3β inhibitor K313 at a higher dose could almost halve the amount of CD80, CD86, and CD40 of DCs, compared with the vehicle group in this study. Nevertheless, another study has indicated that a specific GSK-3β inhibitor combined with LPS could stimulate the maturation of immature BMDCs (Alessandrini et al., 2011), this may be due to different cell purification methods and sources of DCs.

IL-10 could induce a long-term antigen-specific anergic state in CD4+ T helper cells and inhibit them to differentiate into Th1/Th17 cells, so this suppressive cytokine was vital to control inflammatory levels in EAE (Gabrysova et al., 2009). In contrast, IL-12 has a positive effect on the differentiation and expansion of pathogenic Th1 cells in EAE mice (Trinchieri, 2003), while decreased IL-12 secreted by DCregs plays a key role in the expansion of Tregs (Sonobe et al., 2012). Ono et al. have suggested that BMDCs treated with a GSK-3β inhibitor (SB415286) secrete increased IL-10 and decreased IL-12 upon LPS stimulation (Ono et al., 2007). Another study found that syk deficiency in LPS-stimulated BMDCs can increase the amount of IL-10 and decrease the level of IL-6 together with TNF-α through the enhanced phosphorylation of GSK-3β (Yin et al., 2016). As expected, our research also showed that K313, a potential GSK-3β inhibitor, would gain an increased ratio of secreted IL-10: IL-12 in LPS-stimulated BMDCs, compared with the vehicle control. In addition, IL-6 was involved in the Th1/Th2 balance and mediated the polarization of Th17 cells (Bettelli et al., 2006; Darden et al., 2016). Serada *et al.* found that the IL-6 blockade can ameliorate the development and severity of disease in EAE mice by inhibiting the expansion of myelin antigen-specific Th1 and Th17 cells *in vivo* (Serada et al., 2008). Interestingly, another study suggested that a deficiency of CD40 expression leads to a lack of production of IL-6 in a selective inhibitor of GSK-3β-treated DCs (Ono et al., 2007). Our previous study suggested that K313 has the potential to suppress the production of IL-6 and TNF-α in LPS-stimulated RAW264.7 macrophages (Zhao et al., 2018). Martin *et al.* also found that the GSK-3 inhibitor could inhibit the secretions of IL-12, IL-6, and TNF-α in human monocytes (Martin et al., 2005). Our study obtained similar results, suggesting that the expression of CD40 was downregulated by nearly half, accompanied by a significant decrease of IL-6 secretion in K313-treated BMDCs. It is promising that the decreased IL-6 induced by K313 could help inhibit the expansion of self-reactive T cells and control auto-reactive inflammation. In conclusion, K313-treated BMDCs with decreased pro-inflammatory cytokines and elevated immunosuppressive cytokine IL-10 have the potential to inhibit the expansion of pathogenic Th1/Th17 cells and promote the expansion of Tregs.

The decreased expansion of antigen-specific T cells regulated by DCregs is critical in the control of autoimmune diseases (Piemonti et al., 2000). Several studies have suggested that the inhibition of antigen-specific T cells can ameliorate disease severity in EAE mice (Serada et al., 2008; Yoshida et al., 2013; Xie et al., 2017). In this study, the co-culture experimental results confirm the conjecture that K313-modified DCregs significantly suppress the expansion of OVA_323-339_ peptide-specific CD4+ T cells. The above experimental results *in vitro* suggest that K313-treated BMDC has an obvious tolerogenic feature. In order to investigate whether K313-modified DCregs play a therapeutic role in autoimmune diseases, K313-treated BMDCs loaded with MOG_35-55_ were adoptive transferred into the EAE mice. Through disease activity scoring and pathological analyzing, the treatment was found to have a significant positive effect on the EAE disease.

Based on the above results, as a novel benzoxazole derivative, K313 was first found to induce DCregs without cytotoxicity. Moreover, the experiments *in vivo* show that K313-modified DCregs-loaded antigen peptide can ameliorate disease development in EAE mice. The data indicate that K313-modified DCregs might have the potential to be an efficacious treatment for autoimmune diseases, such as MS.

## Data Availability Statement

All datasets generated for this study are included in the article/**Supplementary Material**.

## Ethics Statement

The studies involving human participants were reviewed and approved by the Ethical Review Committee of Chengdu Medical College. The patients/participants provided their written informed consent to participate in this study. The animal study was reviewed and approved by the Animal Care and Use Committee of Chengdu Medical College.

## Author Contributions

YZ carried out the histological analysis, the animal experiment and drafted the manuscript. XLe carried out the cell culture and flow cytometric analysis. XLu participated in the animal experiment. CM participated in the western blotting and ELISA. QZ participated in the design of the study and performed the statistical analysis. YW and YL conceived of the study, and participated in its design and coordination and helped to draft the manuscript. All authors read and approved the final manuscript.

## Funding

This study was supported by the National Natural Science Foundation of China (no. 81871300, no. 81901521), the Sichuan Science and Technology Program (no. 2018jy0481, no. 2018JY0440), Research Fund of Development and Regeneration Key Laboratory of Sichuan Province (no. SYS16-002), Scientific Research Fund of Sichuan Provincial Education Department (no. 18ZA0143) and the Research Fund of Chengdu Medical College (no. CYTD18-01, no. CYZ19-09).

## Conflict of Interest

The authors declare that the research was conducted in the absence of any commercial or financial relationships that could be construed as a potential conflict of interest.

## Supplementary Material

The Supplementary Material for this article can be found online at: https://www.frontiersin.org/articles/10.3389/fphar.2019.01579/full#supplementary-material

Supplementary Figure 1 The survival of K313 treated murine DCs after 48 h of LPS stimulation.

Supplementary Figure 2 The clinic score of EAE mice injected with PBS-treated BMDCs.
