# Supplementary figures and images for: Regulatory Dendritic Cells Induced by K313 Display Anti-Inflammatory Properties and Ameliorate Experimental Autoimmune Encephalitis in Mice
Source: Front Pharmacol. 2020 Jan 28;10:1579. doi: 10.3389/fphar.2019.01579 (PMC6997778; doi:10.3389/fphar.2019.01579)

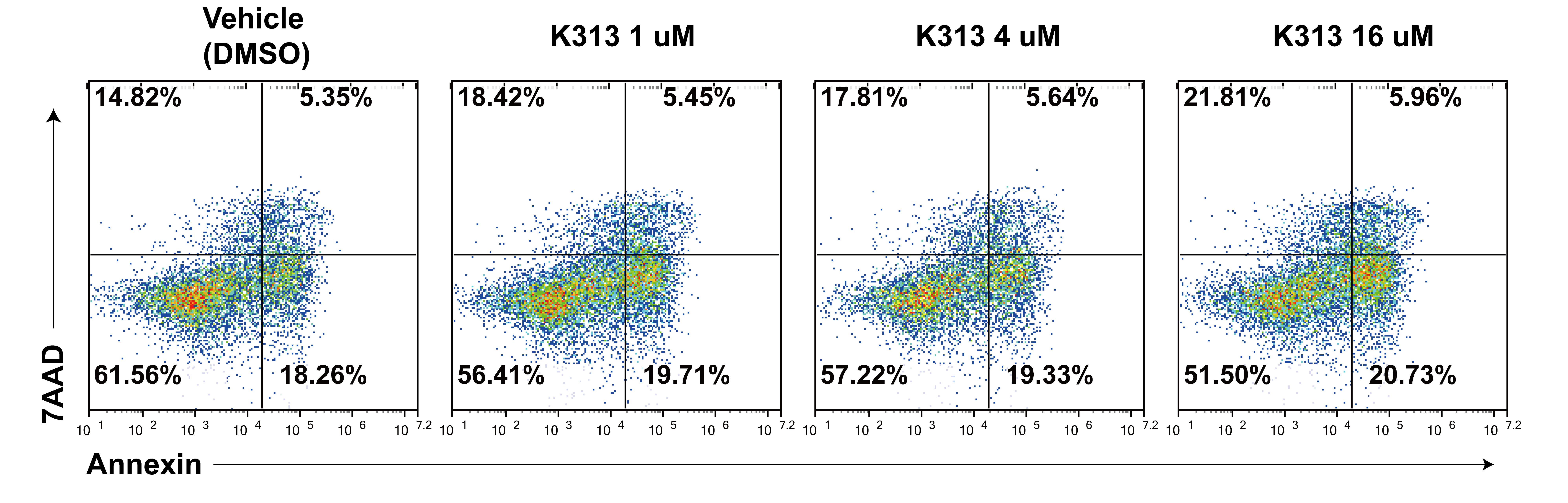

Supplement: Supplementary Figure 1 — The survival of K313 treated murine DCs after 48 h of LPS stimulation. [file Image_1.tif]

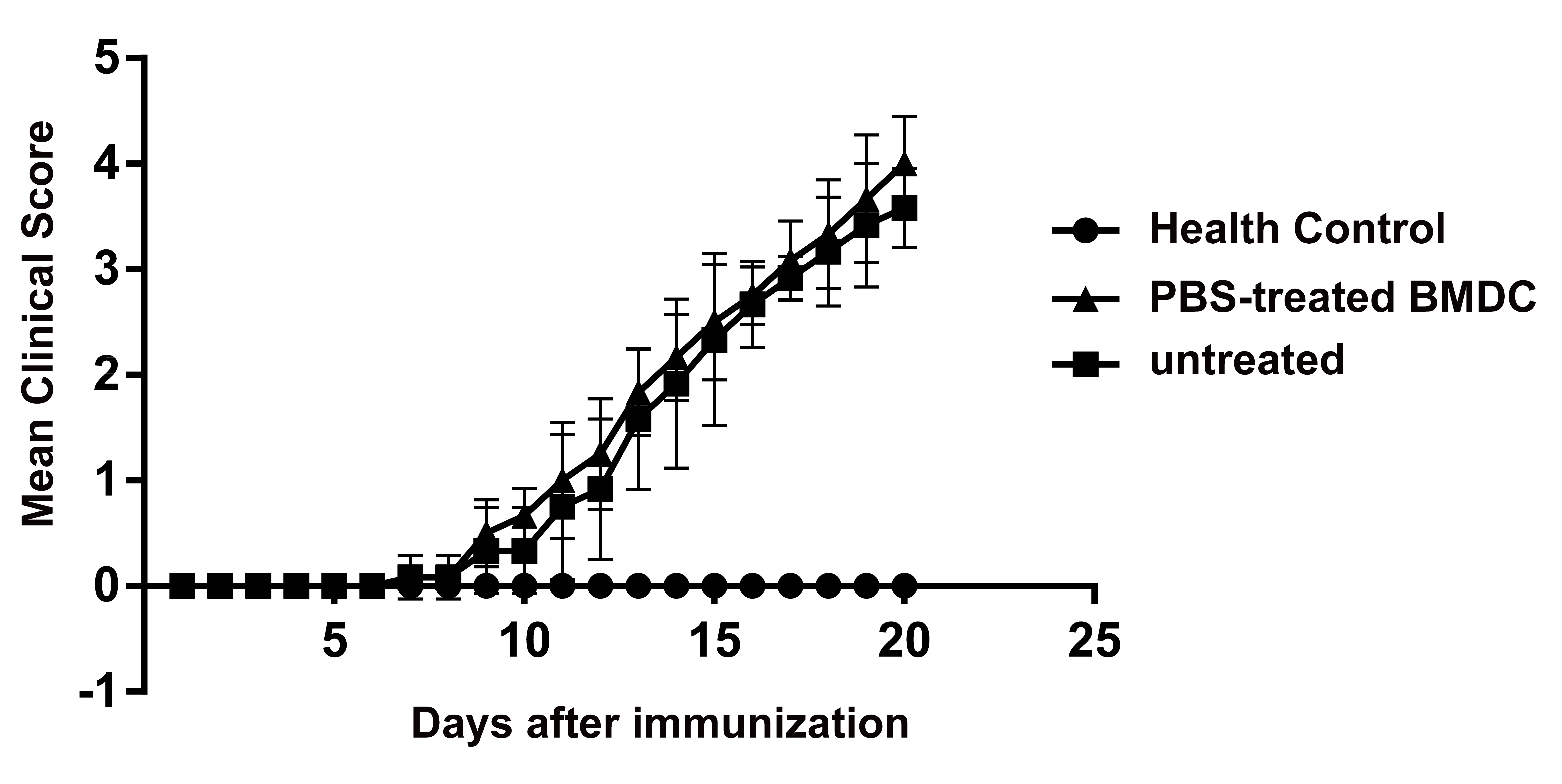

Supplement: Supplementary Figure 2 — The clinic score of EAE mice injected with PBS-treated BMDCs. [file Image_2.tif]
